# Single neurons on microelectrode array chip: manipulation and analyses

**DOI:** 10.3389/fbioe.2023.1258626

**Published:** 2023-09-27

**Authors:** Hongyong Zhang, Pengbo Wang, Nan Huang, Lingrui Zhao, Yi Su, Lingfei Li, Sumin Bian, Mohamad Sawan

**Affiliations:** ^1^ Zhejiang University, Hangzhou, Zhejiang, China; ^2^ Research Center for Industries of the Future, Westlake University, Hangzhou, Zhejiang, China; ^3^ School of Life Science, Westlake University, Hangzhou, China; ^4^ Department of Neurology, Affiliated Hangzhou First People’s Hospital, Zhejiang University School of Medicine, Hangzhou, China

**Keywords:** single-cell manipulation, microelectrode array, dielectrophoresis, electrophysiology, single neurons, network of neurons

## Abstract

Chips-based platforms intended for single-cell manipulation are considered powerful tools to analyze intercellular interactions and cellular functions. Although the conventional cell co-culture models could investigate cell communication to some extent, the role of a single cell requires further analysis. In this study, a precise intercellular interaction model was built using a microelectrode array [microelectrode array (MEA)]-based and dielectrophoresis-driven single-cell manipulation chip. The integrated platform enabled precise manipulation of single cells, which were either trapped on or transferred between electrodes. Each electrode was controlled independently to record the corresponding cellular electrophysiology. Multiple parameters were explored to investigate their effects on cell manipulation including the diameter and depth of microwells, the geometry of cells, and the voltage amplitude of the control signal. Under the optimized microenvironment, the chip was further evaluated using 293T and neural cells to investigate the influence of electric field on cells. An examination of the inappropriate use of electric fields on cells revealed the occurrence of oncosis. In the end of the study, electrophysiology of single neurons and network of neurons, both differentiated from human induced pluripotent stem cells (iPSC), was recorded and compared to demonstrate the functionality of the chip. The obtained preliminary results extended the nature growing model to the controllable level, satisfying the expectation of introducing more elaborated intercellular interaction models.

## 1 Introduction

The importance of a single cell, which forms the basic neural element of life, has received increasing attention in recent years, due to the importance of individual cells on various cellular research, such as single-cell sequencing (Li et al., 2022), cell migration (Yoshida et al., 2016), and cell communication (Wu et al., 2017). Single cell analysis offers several advantages compared to traditional petri dish cell culture, such as its high throughput capacity, the ability to use less sample volume, and the potential to reveal individual differences (Luo et al., 2019). In recent years, various techniques have been reported to manipulate cells, such as dielectrophoresis (DEP) (Chen et al., 2014), magnetic field (Huang et al., 2017), acoustic wave (Yang et al., 2022), and microstructures (Pang et al., 2020) on chips. Farasat et al. (2021) proposed a microfluidic chip to manipulate cells using DEP, created by interdigitated electrodes. Compared with other methods, DEP is a simple technique that offers high efficiency and precision to manipulate single cells, with relatively low cost (Yao et al., 2019). The most quintessential model to study cell communication and collect cellular electrophysiology is culturing network of neurons (NoNs) on MEA chip, recording neural signals by microelectrodes (Zhang et al., 2022).

Human brain’s NoNs are highly interconnected which form functions to process information sequences (Ahrens et al., 2013). Given the increasing number of people affected by neural disorders and the lack of effective therapies, gaining a deep understanding of the mechanism of the human brain has become a top priority (Golyala and Kwan, 2017; Tang et al., 2017). Precise knowledge of NoNs in the human brain is pivotal in comprehending the pathology of neural diseases (Zhang et al., 2022). Conventional methods of studying the human brain, such as electroencephalogram signal recording (Kassab et al., 2018) and magnetic resonance imaging (Centeno and Carmichael, 2014), which are widely used in medical domain, are not accurate enough to reflect detailed neural activities. Moreover, the brain complexity renders it impossible for the available nanoimaging techniques to investigate the fine neural-cell interconnections. Invasive monitoring methods, such as intracortical electrocorticogram signals (Dubey and Ray, 2019) and implantable brain-machine interface-based devices (Ji et al., 2022) are more accurate than the non-invasive or *in vitro* methods; however, these methods exhibit certain limitations, such as individual variation, ethical and safety issues, and incapableness of batch research. In this case, establishing *in vitro* NoN models has become a promising method for investigating human brain diseases (Holloway et al., 2021).

The growth of iPSC induced NoNs on MEA chip for *in vitro* evaluation is becoming an increasingly attractive method for analyzing the brain structure, as it involves fewer ethical issues and doesn’t require complex and large resources for its implementation (Saiding et al., 2022). Microelectrodes can be used for recording neural signals and stimulating neurons through low-intensity currents (Abbott et al., 2020; Kumar et al., 2020; Liang et al., 2021). Recent advancements in state-of-the-art nanofabrication techniques have led to the proposal of various elaborated MEAs in recent years, including large-scale (Tsai et al., 2017), biomimic (Wijdenes et al., 2016), and three-dimensional MEAs (Morales-Carvajal et al., 2020; Xu et al., 2021). Combined with elaborate microfluidic devices (Konishi et al., 2015; Haque et al., 2022), several neural models have been established to study certain specific functions (Osaki et al., 2018; Kajtez et al., 2020; Pelkonen et al., 2020), providing promising platforms to perform *in vitro* analysis of NoNs. However, in most of these studies, the focus was on the signal transmission in NoNs but not in a single neuron, which forms the basic unit of NoNs in the brain (Choi et al., 2021). Moreover, a significant number of neural cells fail to establish contact with microelectrodes, resulting in the inability to record their electrophysiology simultaneously. This low yield is a significant drawback of existing methods of mapping NoNs. Compared to large-scale NoNs on MEAs, small-scale NoNs or even single neuron on MEAs will facilitate us to a deeper understanding of basic neuron functions (Yoshida et al., 2016). Moreover, increasing evidence has validated the importance and ability of single-neuron analysis (Gupta et al., 2020). Therefore, single-cell manipulation for single neuron isolation can aid in precisely locating one cell on one electrode, forming multiple single neuron-based NoNs.

**FIGURE 1:**
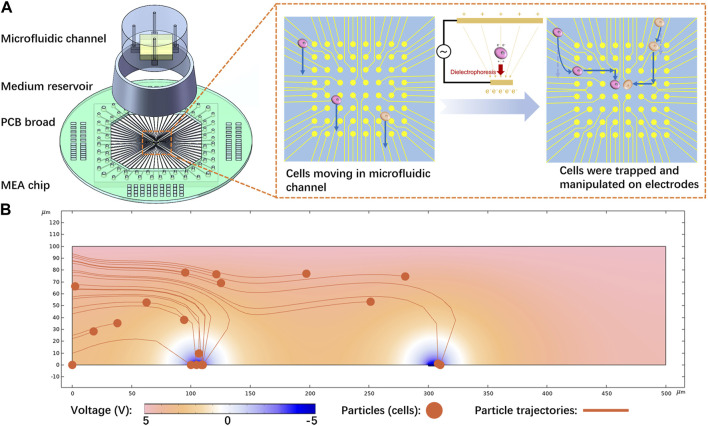
Cell manipulation principle and device: **(A)** Proposed device, primarily comprising a microelectrode array (MEA) chip with a microfluidic structure. Cells are trapped and transferred between electrodes to achieve exact manipulation, **(B)** Simulation results of the cell trapping process. Cells were released from the left side, moving at a flow rate of 10 μm/s. Subsequently, they were subjected to p-DEP and attracted by the electrodes.

In this study, we designed and fabricated a small-scale MEA chip with 8 × 8 electrodes to manipulate single cells and investigate intercellular connections *in vitro*. A non-uniform electric field was produced in microfluidic channels to trap cells. This precise location of cells aided in monitoring their growing process for studying intercellular interactions at real time. Neurons and cancer cells were both manipulated and cultured on the chip. Considering that culturing and observing cancer cells or neurons often requires weeks or even longer periods, a custom medium reservoir was developed for long-term cell culture. Furthermore, a printed circuit board (PCB) was designed and implemented to connect the MEA to a signal generator and a data acquisition system for cell manipulation and cellular electrophysiology recording, respectively. Figure 1A depicts the simplified illustration of the proposed device.

## 2 Results

### 2.1 Working principle of cell manipulation chip

During the experiment, positive-DEP (p-DEP) was used to manipulate cells, as depicted in Figure 1A. A non-uniform electric field was generated by applying voltage between two conductive plates with different sizes. Microelectrodes on the chip were used as the small plate, and a piece of Indium Tin Oxide (ITO) glass inserted in the microfluidic channel served as the large plate. Cells were pumped into the microfluidic channel between the two plates and then subjected to DEP.

COMSOL was used to model and simulate the cell manipulation process and identify the approximate parameters, as illustrated in Figure 1B. Yellow particles representing cells were released from the left side portion and flowed at a rate of 10 μm/s. The microelectrode strongly attracted the cells, which verified the feasibility of the proposed method. According to Eq. 1 (in Section 4.1), decreasing the permittivity of the solution increased the DEP effect. Meanwhile, considering the requirement of maintaining the osmotic pressure balance of the cells, sucrose solution was selected as a suspending solution to manipulate the cells during the entire process, as deeply explored in Section 4.3. Further investigation was carried out on the effect of voltage on DEP and cell trapping (Supplementary Figure S1). The results demonstrated that increasing the voltage expanded the range of dielectric electrophoresis force and captured a larger number of particles. In order to capture one single cell per electrode, we applied smaller voltages for cell manipulation in the subsequent experiments.

### 2.2 Design and fabrication of cell manipulation chip

#### 2.2.1 MEA chip

**FIGURE 2:**
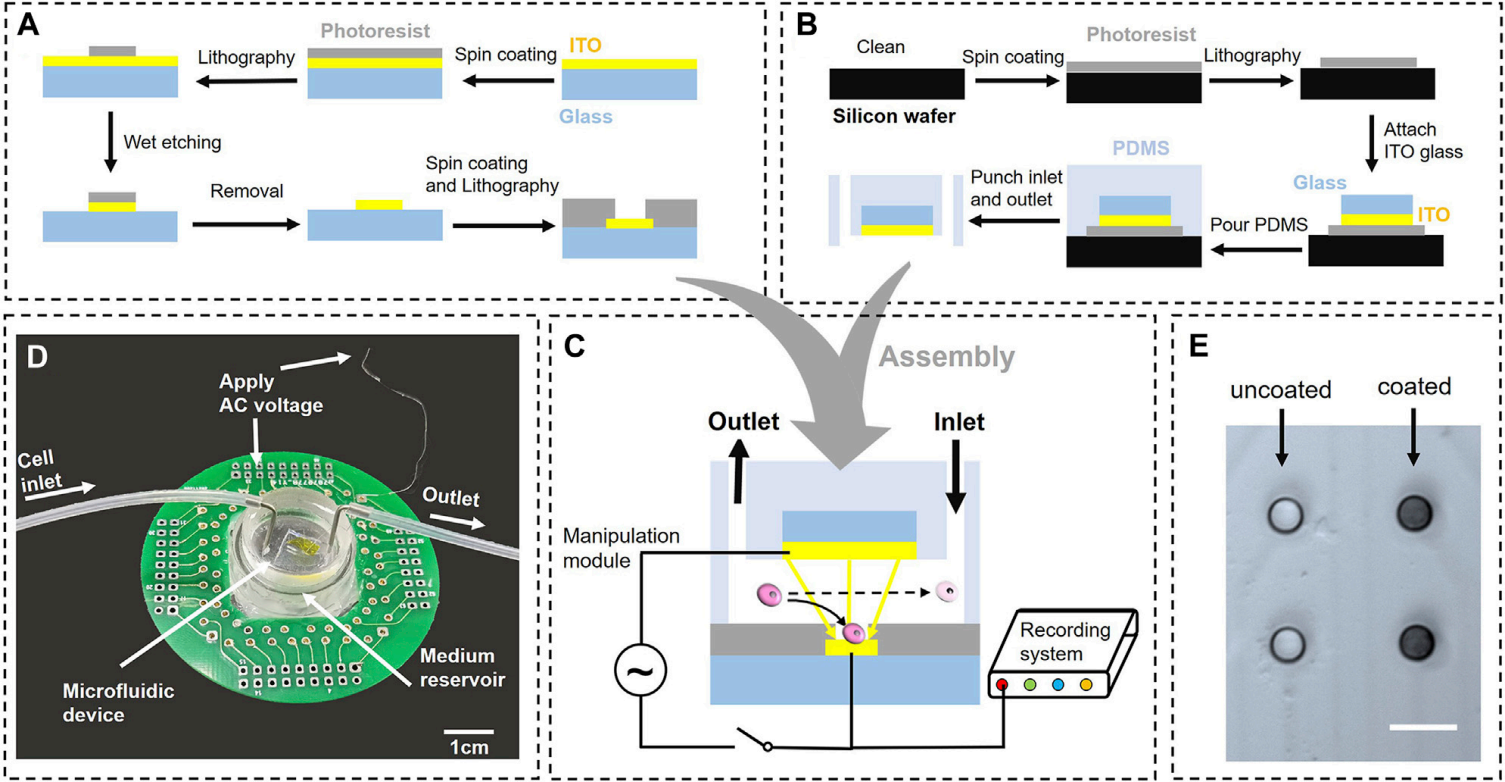
Fabrication and assembly of the entire device: **(A)** Fabrication process of the microelectrode array (MEA) chip. Standard lithography technique was used to pattern the Indium Tin Oxide (ITO) and obtain the insulating layer, **(B)** Fabrication process of the microfluidic device, **(C)** Photograph of the entire device, including the MEA chip, printed circuit board (PCB), and microfluidic device. Cells were pumped into the chip through a rubber tube and manipulated on MEA, **(D)** Assembly and connection of the MEA chip, **(E)** Microelectrodes with and without PEDOT:PSS coating (Scale bar = 20 µm).

The MEA chip facilitated two functions: first, it functions as electrodes for cell trapping, and second, it enables the direct recording of electrical and other signals after processing. Consequently, it serves as an ideal platform to establish single-cell-based NoNs with the function of signal acquisition. For better observation, the MEA chip was manufactured using an ITO glass, owing to its high transparency and adequate conductivity. Compared to traditional metal electrodes, such as gold or platinum, ITO is far more transparent, allowing for better optical viewing. Also, ITO is more affordable and readily available than many electrode materials. Although ITO has higher impedance than gold, we applied a conductive polymer PEDOT: PSS coating to the surface to lower the impedance and improve the electrode-cell interfacing. Figure 2A depicts the fabrication process, which can be summarized as follows: 1) The ITO glass was cleaned using acetone and isopropanol separately for 10 min each in an ultrasonic cleaner; 2) A layer of AZ 1518, a type of positive photoresist, was spin-coated on the glass surface by a spin coater and baked at 95°C for 1 min on the hot plate; 3) A chromium mask was used to pattern the photoresist via lithography; 4) An acid solution containing 18% HCl and 0.5% HNO_3_ was used to, etch ITO at 60°C. Depending on the ITO thickness, the etching time was maintained within 2 min to prevent damage to the photoresist and pattern; 5) The chip was dipped in acetone for 2 h and cleaned using a strong airflow to remove the remaining photoresist; 6) Finally, another layer of negative photoresist (SN 1305) was patterned by repeating the aforementioned steps; this served as an insulating layer that prevented interference. Thus, microwells were fabricated on the electrodes to capture the cells on the electrodes. Four-reference electrodes were placed around the microelectrodes for data acquisition (Supplementary Figure S2). In addition, our fabrication process is scalable and can be implemented with AutoCAD mask design.

#### 2.2.2 Microfluidic device

A microfluidic device was used to maintain a stable flow and generate a non-uniform electric field. Figure 2B depicts the fabrication process of the microfluidic device. A negative photoresist (SU-8 2050) was used to build the mold of the microfluidic channel on a silicon wafer, following the standard lithography technology. Subsequently, a small piece of ITO glass was placed on the photoresist as a large conductive plate. Homogeneously mixed polydimethylsiloxane (PDMS) was poured on the wafer and baked at 60°C for 2 h, followed by cutting the solidified PDMS into suitable shapes using a scalpel. Finally, the PDMS puncher was used to punch the inlet and outlet at the two microchannel ends. The cells were pushed using a syringe pump and flowed into the chip through a rubber tube.

#### 2.2.3 Assembly of printed circuit board (PCB) and device

PDMS and the MEA chip were cleaned using plasma before being gently attached to each other (Figure 2C). The distance between the microelectrodes and conductive plate was identical to the thickness of the photoresist, which was 100 µm. They were connected to a cell manipulation module and signal recording module, controlled by a switch. During the manipulation step, electrodes were connected to the cell manipulation module, which served as a signal generator. This was used to apply a voltage between microelectrodes and conductive plate to generate a non-uniform electric field for cell manipulation. After the cells were settled, the switch turned to the signal recording module to record the neural signals. Considering the challenges in directly connecting ITO glass with wires or pins, a PCB matched with the MEA chip was designed for an alternative operation. A silver conductive gel was used to connect microelectrodes with pins on PCB and attach the chip to PCB. Initially, the pads on ITO and pins on the PCB were aligned by the calibration platform. Subsequently, moderate sliver conductive gel was smeared on their interfaces. They were placed on the hot plate and heated to 200°C for 1 h to dry the gel. Finally, an ohmmeter was used to detect the connection between the pads and pins.

Furthermore, a suitably sized medium reservoir, composed of PDMS, was attached to the chip in the peripheral of the microfluidic device. The medium reservoir was fabricated using a 3D printed mold. To bond the reservoir to the MEA chip, they were first cleaned with acetone in an ultrasonic cleaner and then dried with nitrogen. Next, the contact surfaces were activated by treating them with oxygen plasma for 5 min simultaneously. Then, the plasma-treated samples were promptly bonded with the activated surfaces facing each other. To further strength the bond, the coupled samples were heated at 120°C on a hot plate for 5 min. This measure was taken to minimize the risk of any potential leakage issue; for instance, the solution leaked from the microfluidic channel would be contained in the reservoir without affecting the circuit connections. After the cells adhered to the chip, the microfluidic device was removed and we could culture cells in the medium reservoir, just like the cell culture process in a conventional dish. Figure 2D illustrates the actual image of the completed device. The interface of the inner ring on the PCB was connected to the MEA chip via conductive gel, and the interface of the outer ring was connected to the signal generator or data acquisition system for trapping cells or recording signals.

#### 2.2.4 PEDOT:PSS coating

To improve the quality of neural recording and facilitate cell manipulation, the electrodes were coated with the conductive polymer PEDOT:PSS to reduce impedance and minimize the modulus mismatch between the MEAs and neurons. Figure 2E clearly demonstrates a noticeable difference between the coated and uncoated electrodes on the same chip. The impedance was measured (Supplementary Figure S3) and demonstrated a significant reduction after the PEDOT:PSS coating. This reduction in impedance was sustained for more than 2 weeks.

### 2.3 Investigation of factors influencing the cell manipulation

To obtain optimal results, we conducted an initial investigation into the factors that influence cell manipulation and determined the optimal working conditions. The primary factors that affect cell manipulation included the diameter of microwells and cells, the depth of microwells, and the amplitude of the voltage.

#### 2.3.1 Size of microwells and cells

**FIGURE 3:**
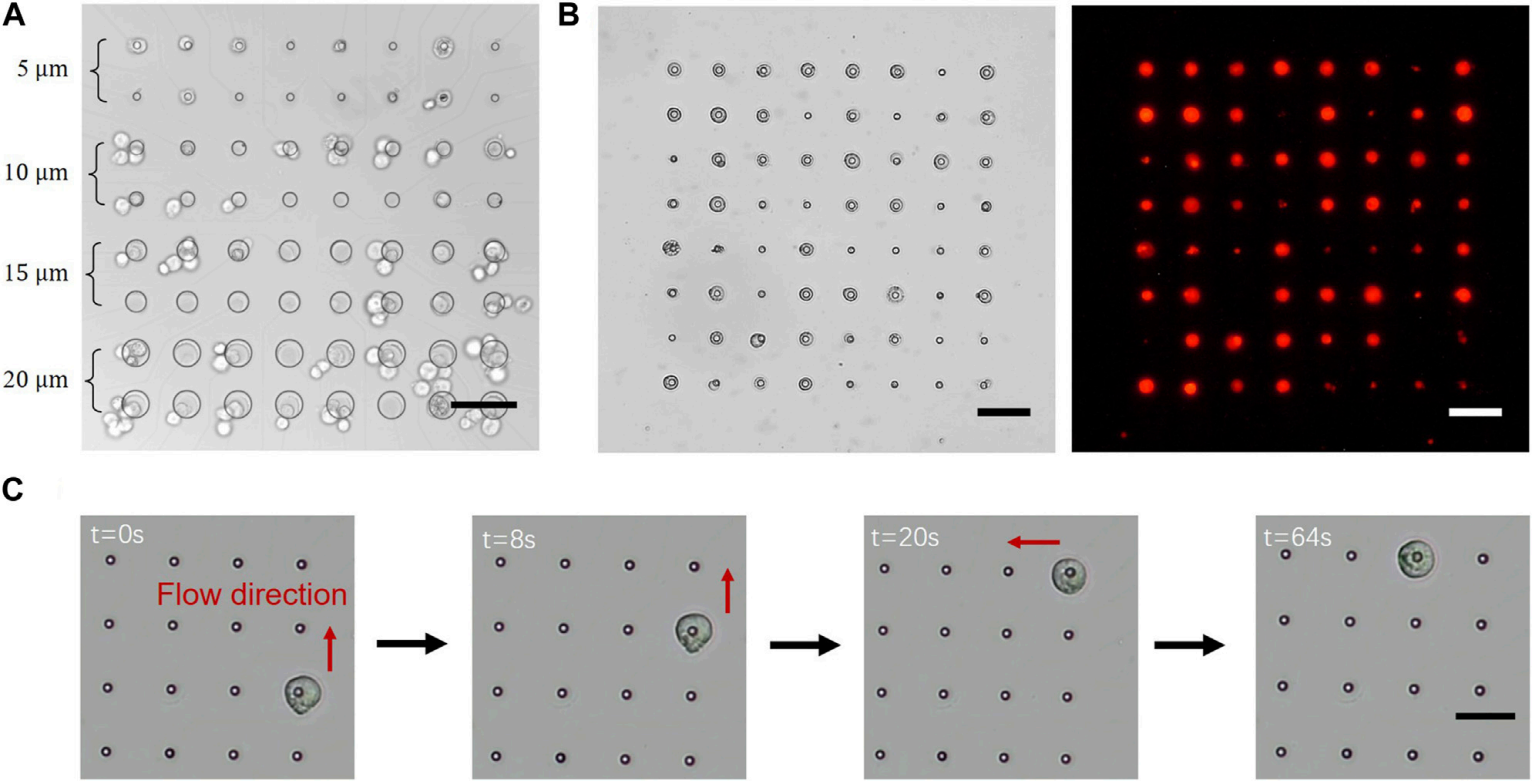
Cell manipulation on the microelectrode array (MEA) chip: **(A)** Cells were trapped by varying the diameters of microwells from 5 to 20 µm on one chip. Most of the microwells on the electrodes trapped cells easily; however, only 5-µm-wells trapped single cells rather than multiple cells; **(B)** 293T cells were trapped on all the electrodes (*left*) and labelled using fluorescence to visualize the captured cells (*right*); **(C)** A cell was precisely controlled to move freely between electrodes. A single cell was randomly trapped in a single electrode and released by reducing the voltage applied to that electrode. The cell moved with the flow and was trapped by the subsequent electrode. (Scale bar = 50 µm).

The size of the microwells determined the area of electrodes involved in the electric field. Although the size of the electrodes was slightly larger than that of the microwells, the area covered by the photoresist insulated the electric field. We fabricated a gradient size of microwells with diameters (D) ranging from 5 to 20 µm to investigate the most suitable size for single-cell trapping (Figure 3A). 293T cells were pumped into the microfluidic channel for tests. Sinusoidal signal with 3 Vpp and 2 MHz frequency were applied between the microelectrodes and conductive plate inserted in the microfluidic channel. Most of the electrodes trapped cells via DEP; however, only 5-µm-microwells could trap single cells as required. In the case of microwells with a diameter larger than 10 μm, two or more cells were trapped on the electrodes. Considering that the average diameter of 293T cells was approximately 10 μm, we determined that the diameter of the microwells should be approximately half that of the cell to achieve single-cell trapping.

The size of cells was also an important factor affecting the amplitude of DEP. According to the equation of DEP described in Section 4.1, the amplitude of the force was proportional to the third power of the diameter of the particle. The larger the cells, the lesser the voltage required to trap them. Nevertheless, the hydraulic resistance increased with the increase in the cell diameter. For instance, as the size of 293T cells was larger than that of neural cells, the minimum voltage required to trap 293T cells was less than that required to trap neural cells. However, under the same flow speed, 293T cells were easier to be pushed away, indicating that a larger voltage was required to attach the cells to the electrodes. Collectively, the suitable voltage for different cell types may vary, as it is influenced by several factors including the diameter of cells and flow rate.

#### 2.3.2 Depth of the microwells

Microwells of an appropriate depth prevented the trapped cells from being pushed away by the flow. Excessively deep microwells caused cells to become entirely trapped within the wells, preventing them from communicating with other cells and hindering the formation of NoNs and precise manipulation. The optimal depth of microwells was determined to be slightly smaller than the radius of cells at approximately 4 µm. A surface profile measuring system was used to measure the depth of the wells, as depicted in Supplementary Figure S4. The depth of the fabricated microwells was approximately 4 μm, which did not affect cell manipulation and communications.

#### 2.3.3 Amplitude of the voltage

The amplitude of the voltage signal was pivotal in controlling the magnitude of DEP during the manipulation process. Insufficient voltage resulted in cells trapping failure, while excessive voltage caused two or more cells to be trapped on a single electrode. Furthermore, the application of a high voltage damaged or even destroyed the cells, thereby affecting the formation of NoNs. To identify the critical voltage for cell trapping, the voltage amplitude was initiated from 0.1 Vpp and gradually increased until the cells were trapped on the electrodes. Considering that all electrodes were controlled independently, if two or more cells adhered to one electrode, the voltage on that electrode alone was reduced without affecting the other electrodes.

### 2.4 Cell manipulation

To reduce the risk of infection, all components and devices were washed with 75% ethanol and exposed to ultraviolet light overnight prior to the experiment. The manipulation buffer was filtered with a 0.22 µm filter and pumped into the microfluidic channel in advance to infiltrate the surface and prevent the formation of bubbles. Following the protocol of neurons culture, the surface of MEA chip was coated with a layer of Poly-L-ornithine hydrobromide/Laminin to enhance cell adhesion ability. First, inlet 15 μg/mL Poly-L-ornithine hydrobromide solution in phosphate-buffered saline (PBS) into the microfluidic channel and incubate at 37°C and 5% CO_2_ for 2 h. Second, wash the channel by PBS twice and inlet 5 μg/mL laminin in DMEM/F-12 into the channel, following by another 2 h of incubation. Subsequently, cells were gently suspended in the manipulation buffer at $5\times 10^{4}$ cells/mL and loaded in a plastic syringe. The syringe was then installed on a mechanical pump, and the cells were pumped into the microfluidic channel at 10 μL/min. Simultaneously, the microelectrodes and conductive plate were connected to a signal generator, and the voltage was set to 0.1-Vpp-sine wave at 2 MHz. Once the flow was stabilized, the voltage was applied to the chip and cell manipulation was initiated.

#### 2.4.1 Trapping of single cells

The first step in cell manipulation involved the trapping of single cells onto the microelectrodes. Fluorescence-labelled 293T cells were pumped into the device and used for the manipulation test. The applied voltage was initiated from 0.1 Vpp and gradually increased until cells were trapped on the microelectrodes, which was generally at 0.6 Vpp. At times, cells that had been trapped on microelectrodes became detached due to the flow. In such cases, the voltage could be increased or the flow rate could be decreased to prevent cell movement. Once the majority of the electrodes had trapped single cells, a clean manipulation buffer without cells was introduced into the chip to remove any excess cells, while those cells trapped by the electrodes were retained (Figure 3B).

#### 2.4.2 Precise positioning of single cells

After being trapped onto the microelectrodes, cells were precisely manipulated into specific positions by transferring cells between electrodes (Figure 3C). At the beginning of the process, the voltage applied to the electrode on which the cell was trapped was disconnected to release the cell, which moved with the flow and was trapped by the subsequent electrode. The cell was repeatedly subjected to this operation until it reached the goal electrode’s line. Afterwards, the flow direction was altered to vertical, and the identical procedure was repeated. Supplementary Video S1 showed the process of cell manipulation in two directions, because the microfluidic channels were cross-shaped. Owing to these x- and y-direction deliveries, cells could be manipulated toward any electrode on the chip. Also, more than one cells could be simultaneous manipulated on the MEA chip since the electrodes are independently controlled. However, cells can only be moved concurrently in the same direction, one step at a time, as shown in Supplementary Video S2. After the cell was positioned correctly, the flow was stopped for 30 min to allow the cell to adhere to the electrode. The culture medium was gradually pumped into the chip until the manipulation buffer was completely displaced. The device was placed in a 37°C 5% CO_2_ incubator for 8 h, and the single cell was allowed to strongly adhere to the electrode. The microfluidic device was gently removed, and a fresh culture medium was added to the medium reservoir. Finally, the single cell was cultured in the medium reservoir, like cell culture in a conventional culture dish.

### 2.5 Cell damage caused by the electric field

**FIGURE 4:**
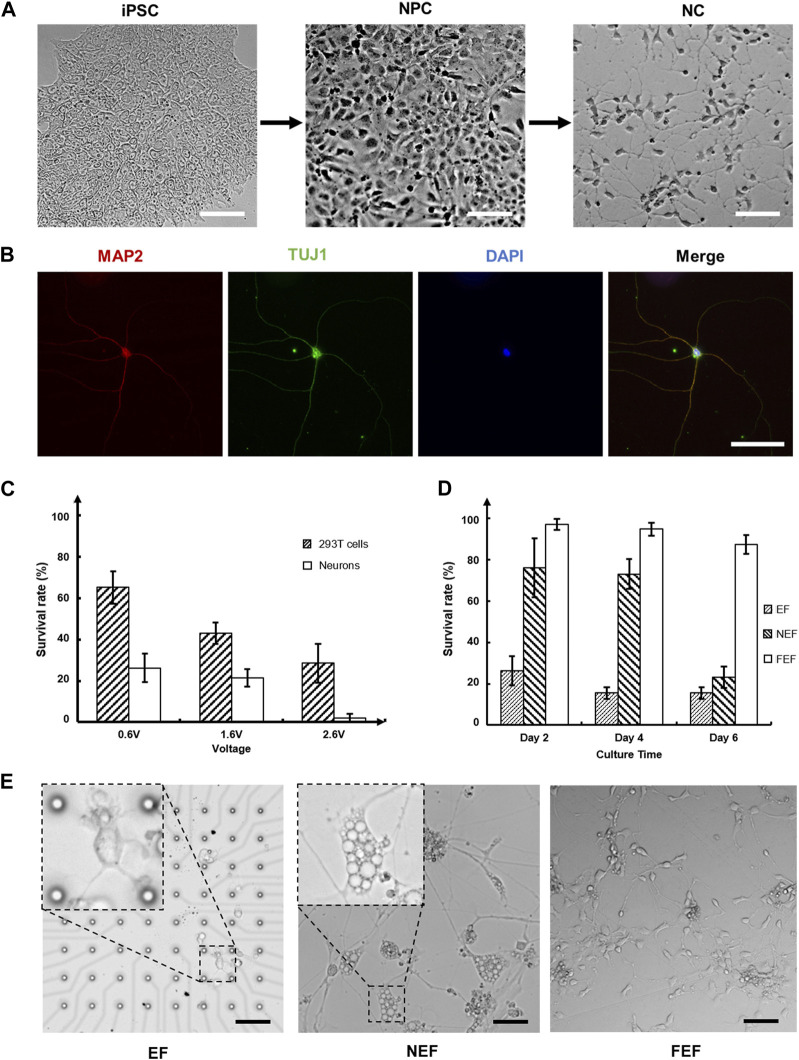
Neural cells culture, validation, and stimulation on chip: **(A)** The process of differentiation from induced pluripotent stem cells (iPSCs) to neural progenitor cells (NPCs) and subsequently to neuron cells (NCs). Each step requires approximately 3 weeks to grow (Scale bar = 50 µm); **(B)** The immunofluorescence staining of neurons. Visualizing the morphology of neurite outgrowth through the detection of microtubule associated protein 2 (MAP2) in red, class III beta-tubulin (TuJ1) in green, and 4′,6-diamidino-2-phenylindole (DAPI) in blue, confirmed the maturation of neurons (Scale bar = 50 µm); **(C)** 293T cells and neurons were stimulated under different voltages for 5 min. Cell survival rates were evaluated after 24 h of culturing; **(D)** Neurons were stimulated at 0.6 Vpp for 5 min. The survival rates of neurons in three distinct areas were assessed on days 2, 4, and 6, with a significant decrease observed in the near electric field (NEF) area on day 6; **(E)** The morphology of neurons in **(D)** on day 6. Neurons in the NEF area developed multiple symptoms, such as swelling, severe cytoplasmic vacuolization, and plasma membrane blebbing, indicating oncosis, a specific mode of cell death. The neurites of neurons in the electric field (EF) area were thinner and shorter, likely due to the damage caused by the electric field. The neurons in far electric field (FEF) area were healthy, almost unaffected by the electric field. (Scale bar = 100 µm).

To investigate the effects of the electric field on cells, we tested the cell survival rate by stimulating both 293T and neural cells under different voltages. The neurons used in this study were induced from human induced pluripotent stem cells (hiPSCs), which is closer to neurons in human body than primary neurons from rats (Figure 4A). To evaluate the function of neurons, microtubule associated protein 2 (MAP2) and class III beta-tubulin (TuJ1) were used as neuron-specific cytoskeletal protein markers, DAPI (4’,6-diamidino-2-phenylindole) was used as the standard cell nucleolus marker to stain a single neuron. The immunofluorescence staining result images were captured using DeltaVision Ultra, as shown in Figure 4B. The detection of MAP2 (red), TuJ1 (green) and DAPI (blue) demonstrated the clear morphology of the neurite outgrowth and indicated the maturation of the neurons. To further observe the morphology of the neurons, they were fixed by glutaraldehyde and captured under scanning electron microscope (SEM), as shown in Supplementary Figure S5.

A sinusoidal voltage with a 2-MHz-frequency was used to stimulate cells for 5 min, and cell survival rates were evaluated after 24 h (Figure 4C). The distance between the microelectrodes and conductive plate was 100 μm, which matched the height of the microfluidic channel. As the minimum voltage required for cell manipulation was 0.6 V, 65.25% ± 7.76% (*n* = 4) of the 293T cells remained alive after manipulation; the cells were normally reproduced. With the increasing voltage, the survival rate decreased significantly. In comparison to 293T cells, neural cells were more sensitive and intolerant to the electric field, with only 26.27% ± 6.90% (*n* = 4) of neurons surviving after 0.6 Vpp stimulation. Nearly all neurons were dead under 2.6 Vpp stimulation, indicating that it was the maximal voltage suitable for neuron manipulation. The results presented above clearly demonstrated that neurons had a significantly electrical tolerance compared to cancer cells.

According to the equation, the value of DEP response is determined by the gradient of the electric field, which relies on factors, such as voltage, distance between electrodes, and the actual spatial distribution of the electric field. In our microfluidic device, the distance and spatial distribution remained constant, leaving voltage as the sole adjustable parameter to influence DEP. So, we used voltage as a representation of the force, to quantify the value of DEP force versus the flow rate used in this study. We set the voltage to a specific value, and slowly increased the flow rate to determine the critical flow rate at different voltages, as shown in the Supplementary Figure S6A. We found that increasing voltage, and thereby DEP force, resulted in an increase in the critical flow rate. A trapped cell was subject to several forces, including flow shear stress, DEP force, support force and intermolecular force, as shown in Supplementary Figure S6B. Normally, these forces were balanced and keep the cell on electrodes. However, when the voltage is above 6 V and flow rate above 1,021 ul/min, cells couldn’t withstand these forces and are torn apart. The top half of the cell was suddenly teared up and disappeared with flow. After stopping the flow, we could observe residual cell debris on the electrodes, representing the lower half of the cell. Due to the damage of electric field on cells, we should set voltage as low as possible, usually low than 1 V.

An intriguing phenomenon was observed when exploring the impact of the electric field’s relative location on neurons during the experiments. The size of the medium reservoir was designed to be larger than that of the conductive plate, ensuring that only the cells located at the center of the MEA chip were stimulated; this specific region was defined as the electric field (EF) area. Neurons near the conductive plate, referred to as the near electric field (NEF) area, were also indirectly affected by the electric field. Neurons located in the outskirts, also known as the far electric field (FEF) area, remained almost entirely unaffected by the electric field. Figure 4D depicts the evaluated cell survival rates for these areas after 5 min of stimulation at 0.6 Vpp on three separate days (days 2, 4, and 6). Approximately 25% of the neurons remained alive in the EF area, similar to the previously obtained result. Neurons located in the FEF area, where the impact of the electric field was minimal, demonstrated a survival rate of up to 90% within 6 days. Furthermore, the neurons in the NEF area experienced rapid cell death after the fourth day. To gain further insight into the cells condition in each area, we observed the morphology of neurons on day 6 (Figure 4E). Neurons in the NEF area exhibited swelling, accompanied by additional symptoms of severe cytoplasmic vacuolization and plasma membrane blebbing, indicating a particular mode of cell death known as oncosis, as reported in a previous study (Guan et al., 2018). This observation confirmed that even weak electric field stimulation could affect the morphology of neurons and induce cell death, thus validating that the sharp decrease in the cells survival rate within the NEF area on day 6. This finding further provided additional insight into the reason why neurons in the EF area were more susceptible to this phenomenon than those in the other two areas, as neurites in the EF area were considerably smaller in size.

### 2.6 Spontaneous neural activity recording

**FIGURE 5:**
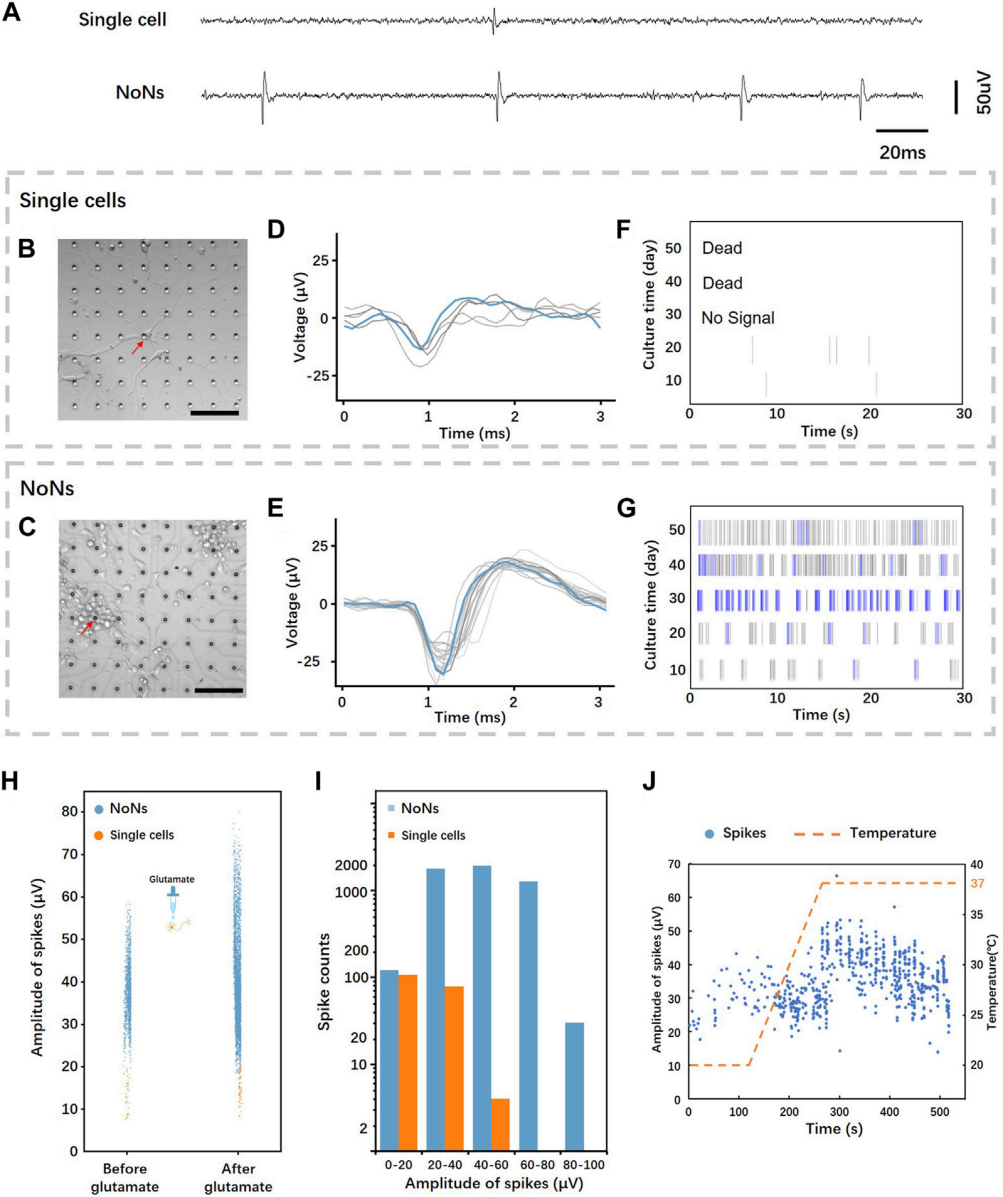
Neural signals recording and analysis from single cells and NoNs: **(A)** Representative field potentials recorded from a single neuron and NoNs; **(B,C)** culture single neurons and NoNs on MEA chip (Scale bar = 100 µm); **(D,E)** Typical overlaid spike waveform from single neurons and NoNs; **(F,G)** Typical raster plot of the spontaneous firing of single neurons and NoNs; **(H)** Comparison of spike distribution from single neurons (orange) and NoNs (blue) before and after glutamate treatment; **(I)** Histograms comparing spike counts and amplitudes between single neurons and NoNs; **(J)** The distribution of spikes in NoNs varied over time as the temperature changed.

Recording neural signals from both single cells and NoNs was conducted to demonstrate the feasibility of capturing neural signals from neurons. Figure 5A shows a fragment of neural signals from single cells and NoNs on day 20. Single cells were manipulated, matured, and recorded on MEA chip as shown in Figure 5B, followed by labelling with fluorescence to display the morphology of single neurons (Supplementary Figure S7A). The growing process of single cell on electrodes was also monitored by microscope in representative stage (Supplementary Figure S7B). Meanwhile, NoNs were cultured on MEA chip without any manipulation and recorded simultaneously, as illustrated in Figure 5C. The chips were connected to a commercial data acquisition system every 2 days, and each recording session lasted for 30 min. Neural signals were successfully recorded from single cells from day 8 to day 34. However, by day 34, all single neurons had died and detached from the MEA chip. The recordings of NoNs began on day 6 and are ongoing at present (more than 80 days). We chose the data from one representative electrodes of single neurons and NoN, pointed by red arrows in the figure, to further analysis the difference of their performance.

The data acquisition system’s companion software was used to analyze the data and its details are outlined in 4.4. The overlaid spike waveform of single cell (Figure 5D) displays an average spike duration of around 1 ms, with amplitude fluctuating during the recording. In contrast, the overlaid spike waveform of NoNs (Figure 5E) differs slightly from that of a single cell with an average spike duration of approximately 2 ms. Furthermore, the amplitude of the NoN’s waveform is also greater and more stable than that of a single cell. The raster plot (Figures 5F, G) exhibits the recording of the spontaneous activity of single cell and NoNs, with the change in days. As shown in the figure, the single cells exhibited activity during the first 20 days but gradually declined until they died, as previously mentioned. Conversely, the NoNs demonstrated increasing activity during the first 30 days, reaching their peak on day 30. Compared to the electrophysiology of single cell, the spikes of NoNs were more frequent throughout the recording. Moreover, many bursts (indicated by blue lines) appeared around day 30, indicating the maturation of the NoNs. Additionally, the spike amplitudes of NoNs increased with culturing time, reaching their maximum peak on day 30 (see Supplementary Figure S8).

To validate that the recorded signals were spontaneous spikes from NoNs, we administered 20 uM glutamate into the culture medium during the recording process on day 20. Glutamate is a major excitatory neurotransmitter in the central nervous system that can enhance the neural activities (DEMİRKAYA et al., 2021). Figure 5H illustrates the response of NoNs (depicted by blue dots) and single neurons (depicted by orange dots) to glutamate. Following glutamate treatment, the firing frequency and amplitude of spikes from NoNs demonstrated a statistically significant increase (average amplitude increased by 25.3%, average frequency increased by 194.5%). In the case of single cells, the response to glutamate was comparable but less pronounced, with an average amplitude and frequency increase by 24.7% and 184.4%, respectively. Furthermore, we recorded the number of spontaneous spikes from three sets of NoNs and single neurons for 15 min on day 24. The results revealed 5,106 spikes from NoNs and 189 spikes from single neurons, as shown in Figure 5I.

The impact of temperature on neuronal electrophysiology was investigated afterwards, as shown in Figure 5J. The neurons were acclimated to room temperature (18°C) for 5 min prior to being connected to the data acquisition system for recording the neural signals from NoNs and single neurons. Following 2 min of recording, the temperature was gradually elevated to 37°C. As the temperature increased, the frequency and amplitude of firing spikes also showed a significant increase, emphasizing the notable effect of environmental temperature on neuronal activity.

## 3 Discussion

We proposed and implemented a prototype single-cell manipulation chip based on MEA using the principle of p-DEP. Compared with the traditional MEA, we could build customized networks of neurons, and every neuron could be recorded. The traditional network of neurons on MEA chip is random, unpredictable, leading to low repeatability. In general, this work combined the ability of single cell manipulation and neurons recording, both for improving electrode-cell coupling and for the possibility of creating customized neuronal networks, recording and stimulation.

We trapped and transferred 293T cells between electrodes to demonstrate the function of the chip and explored various factors influencing cell manipulation, such as the voltage amplitude and the diameter and depth of microwells. We also successfully manipulated the neural cells on the chip and a portion of them remained viable for up to 30 days. Our investigation delved deeper into the effects of electric field on the neurons and 293T cells, revealing that intense electric field stimulation altered the morphology of neurons and triggered cell death. Notably, the electrical tolerance of neurons was significantly lower than cancer cells. The conditions for cells on the chip resemble those during electrotransfection, a biological technique commonly employed in both *in vivo* and *in vitro* studies to deliver molecules into cells through the electric field (Chang et al., 2016; Cervia and Yuan, 2018). Several studies have reported the impact of electric field on neural cells (Heida, 2003). Moreover, various parameters are known to impact the cell survival rate, such as the cell type, the amplitude of the voltage and the duration of stimulation. Utilizing the critical voltage for cell trapping is an effective strategy to increase the cell survival rate. To minimize the damage inflicted on the cells by the external environment, all cell operations were completed within 5 min in this study. We subsequently cultured single neurons on the electrodes and recorded signals from them. In comparison to the electrophysiology from NoNs, single cells exhibited notably weaker physiological electrical activity, as evidenced by their lower amplitude, frequency and quantity, as well as the absence of bursts. These findings suggested that our microfluidic MEA chip has the potential to serve as a valuable platform for developing precise NoN models for *in vitro* studies of intercellular connections among individual cells.

The MEA chip’s simple fabrication allows for the adjustment of all its parameters, including the size of microwells and the positions of electrodes and wells. If other types of cells need to be manipulated, such as Hela cells with an average diameter of approximately 40 μm, the diameter of microwells can be easily adjusted to 20 µm. The distance between microwells, which also determines the distance between the cells, can be precisely controlled, allowing for flexible and precise cell culture density. The design flexibility enables the development of more precise models, such as the neuron-muscle conjunction model where neural cells are arranged to the left and muscle cells to the right for clear observation of their connections.

Our proposed MEA chip can be further improved in three directions. First, the current MEA chip based on ITO glass can only support hundreds of electrodes owing to space limitations, resulting in a small number of neurons on the chip that cannot fully mimic the performance of neurons inside the body, thereby limiting the complexity of functions achieved. To address this limitation, complementary metal-oxide-semiconductor technology can be used to fabricate large-scale MEAs. By integrating control circuits and recording module into the chip, it can be made smaller and more convenient to operate. Second, we acknowledge the negative effect of the electric field on cells, particularly on neural cells during the manipulation process. To address this issue, we intend to improve the design of the structure to further decrease the critical voltage required for manipulation. We also plan to further analyze the mechanism of the observed cell oncosis and identify effective strategies to suppress it. Third, single cells require adequate nutrition and intercellular communication to survive, it is imperative to develop a method for maintaining their viability over time. This can be achieved by introducing culture medium from traditional neurons culture dish to single neuron chip, allowing for a continuous supply of nutrients, as well as facilitating intercellular communication. These improvements will enhance the performance of the MEA chip and expand its potential applications in various fields, including neurobiology and drug discovery.

## 4 Materials and methods

### 4.1 Principle of DEP

DEP was used to trap single cells on microelectrodes for manipulation. Electrically neutral particles in a non-uniform electric field were polarized and pushed by force. The equation of DEP can be expressed as (Yao et al., 2019)

$$F_{DEP}=2\pi {\left(\frac{d_{c}}{2}\right)}^{3}\varepsilon_{f}real\left[CM\left(\omega \right)\right]\nabla {\left|E_{rms}\right|}^{2}\text{ with}$$ (1)

$$CM\left(\omega \right)=\frac{\varepsilon_{c}^{*}-\varepsilon_{f}^{*}}{\varepsilon_{c}^{*}+{2\varepsilon }_{f}^{*}},$$ (2)

where $d_{c}$ denotes the diameter of the cell in the electric field; *E*_*rms*_ indicates the root mean square of the electric field intensity; and $\varepsilon_{c}^{*}$ and $\varepsilon_{f}^{*}$ represent the complex relative permittivities of the cell and fluid, respectively. The sign of $CM\left(\omega \right)$ determined whether the force was positive or negative, indicating the direction of the force. Additionally, the direction of the force was determined by the direction of the electric field intensity rather than that of the voltage. Therefore, we used a high-frequency alternative voltage signal to prevent damaging the cells when manipulating them.

### 4.2 Culture and labelling of cells

293T cells have been widely used in engineering tests owing to their high viability and rapid reproduction. In this study, 293T cells were cultured with Dulbecco’s modified eagle medium (DMEM) high-glucose (Thermo Fisher Scientific, United States), containing 10% fetal bovine serum and 1% penicillin and streptomycin, in a humidified 5% CO_2_ incubator at 37°C. Before manipulating the 293T cells, they were dissociated into single cells using Trypsin (Thermo Fisher Scientific, United States) and collected in a 15 mL tube with DMEM. Additionally, they were stained with fluorochrome Cell Tracker CM-Dil for better observation using the following procedure. The collected cells were centrifuged at 1,200 rpm to remove the supernatant. Then, 293T cells were resuspended in a 1 mL working solution of CM-Dil and incubated at 37°C for 5 min. Subsequently, they were moved to a 4°C refrigerator for 15 min. Finally, the cells were washed three times with PBS and prepared for manipulation after centrifuging and resuspending in the manipulation buffer.

Primary neural cells from rats have been commonly used in previous disease models. However, progressive evidence has shown that molecular mechanisms in animals differ from those observed in human beings (Seok et al., 2013). To better analyze the neural diseases of humans, neurons differentiated from hiPSCs were used to develop the proposed NoNs (Geraili et al., 2018). We followed the protocol of STEMCELL commercial STEMdiff™ SMADi Neural Induction Kit to induce the iPSCs into neural progenitor cells (NPCs) for 18 days. Subsequently, the STEMdiff™ Forebrain Neuron Differentiation Kit and STEMdiff™ Forebrain Neuron Maturation Kit were used for further differentiation and maturation of neuron cells (NCs), respectively. To evaluate the function of neurons, 4% paraformaldehyde was used for a 10-min-cell fixation, followed by two 5-min-PBS washing processes. Additionally, 0.3% TritonX-100 in PBS was used to treat the cells for 30 min, followed by three 5-min-PBS washing processes. The cell sample was then blocked overnight in a block solution [PBS with 3% bovine serum albumin (BSA) and 0.1% TritonX-100] at 4°C. Primary antibody incubation was conducted in the block solution at around 26°C for 2 h, followed by three 5-min-PBS washing processes. Secondary antibody incubation was also performed for 1 h under the same conditions as primary antibody incubation, followed by three 5-min-PBS washing processes. Finally, Fluoromount™ Aqueous Mounting Medium was added to treat the staining sample and seal the coverslips. To capture the images, microtubule associated protein 2 (MAP2) and class III beta-tubulin (TuJ1) were used as neuron-specific cytoskeletal protein markers. Additionally, DAPI (4’,6-diamidino-2-phenylindole), a blue-fluorescent DNA stain which serves as the standard cell nucleolus marker. They were all incubated for 5 min.

The 293T cell line presented in this study was obtained commercially from Sino Biological Inc. (Beijing, China). The induced pluripotent stem cell line presented in this study was obtained commercially from Sanqi Biological Inc. (Shenzhen, China).

### 4.3 Cell manipulation buffer

The simulation results of COMSOL indicated that to achieve p-DEP on the MEA chip, the permittivity of the solution should be lower than that of the cell. The higher the difference in the permittivity, the larger the DEP. Additionally, the osmotic pressure of the buffer should concur with that of the culture solution. Therefore, sucrose (Klamar, Shanghai, China) was used to adjust the osmotic pressure, with the addition of BSA to prevent nonspecific binding. The osmotic pressures of the culture medium and sucrose solutions were measured using an osmometer, as shown in Supplementary Figure S9A. The results aided in determining the recipe of the manipulation buffer for 293T cells [11.2% (w/v) sucrose and 0.5% (w/v) BSA dissolved in deionized water] and neural cells [9.6% (w/v) sucrose and 0.5% (w/v) BSA dissolved in deionized water]. We also measured the permittivity of sucrose solution under different frequency (Supplementary Figure S9B), indicating that the frequency of manipulation voltage should be larger than 10 kHz.

### 4.4 Acquisition and analysis of neural signals

Recordings and analyses of signals from neurons were conducted using the Maestro Pro electrophysiology recording and stimulation system (Axion Biosystems, GA, United States) and its corresponding software. The system allowed simultaneous recording from all electrodes at a sampling rate of 12.5 kHz, with real-time display capabilities. To establish a connection between our MEA chip and the commercial system, a specially designed PCB was employed, as depicted in Supplementary Figure S10. Data was collected using the neural spike setting, employing a gain of 1,000× and a bandpass filter ranging from 200 Hz to 4 kHz. Furthermore, the AxIS software identified spikes as signals with amplitudes above a threshold of 6 standard deviations from the mean noise level, while bursts were defined as a minimum of five spikes with a maximum interspike interval of 100 ms.

### 4.5 Fabrication and characterization of PEDOT:PSS coatings

The standard electroplating process was used for coating, which can be summarized as follows: 1) PEDOT:PSS electrolyte was prepared by mixing 20 mM EDOT (Klamar, Shanghai, China) and PSS (Klamar, Shanghai, China) 0.4 wt% in deionized (DI) water; 2) Cleaning the MEA chip with acetone and oxygen plasma; 3) Connecting the microelectrodes pads with platinum wires using conducting resin; 4) Attaching the working electrode (Pt) to the cathode and the sample to the anode for electroplating. Deposition of the PEDOT:PSS was carried out for 10 min under a voltage of 2 V. It should be noted that the voltage should not exceed 3 V, as this may result in the breakage of the ITO electrodes. An Intan RHX recording and stimulation system (Intan Technologies, Los Angeles, CA) was used to measure the impedance of microelectrodes before and after PEDOT:PSS coating. The electrodes were immersed in 1 ×PBS solution and measured at 1 kHz.

### 4.6 SEM imaging

To prepare the SEM sample, neurons were initially fixed in a stationary liquid containing 2.5% glutaraldehyde (Yuanye Biotechnology, Shanghai, China) and 2% PFA (Yuanye Biotechnology, Shanghai, China) at 4°C for an hour, obtained from Yuanye Biotechnology, Shanghai, China. The sample was then washed thrice with 0.1 M Phosphate Buffer (PB) with pH of 7.3. Subsequently, the neurons were fixed again by 1% osmic acid (in 0.1 M PB) on ice for 1 h and gently washed three times with deionized water. Afterward, the sample was dehydrated by gradient alcohol ranging from 30% to 95%, followed by thrice dehydration with 100% alcohol. Finally, the sample was dried at a critical point and coated with a 5 nm layer of gold on the surface. The SEM observation was made thereafter.

### 4.7 FEM simulation

COMSOL Multiphysics^®^ version 5.5 (COMSOL Co., Ltd., Sweden) was used to simulate the cell manipulation process. The parameter settings for cells were based on a previous study (Chen et al., 2014). The measured parameters of sucrose solution from 4.3 were used during the simulation. Electric current (ec) module was used to simulate electric field distribution; while Creeping Flow (spf) module was used to simulate laminar flow; and Particle Tracing for Fluid Flow (fpt) module was used to simulate dielectrophoresis and the trajectories of cells.

### 4.8 Measurement of surface profile

The spin coater (REESEEN PvS-mini7, Jiangyin J. Wanjia Technology Co., Ltd., China) was used to control the thickness of photoresist, which determined the depth of microwells. A surface profile measuring system (Bruker, Dektak XT, Germany) was used to measure the depth of the wells. The softness of material was set to 5 and scanning speed was set to 5 μm/s. The surface profile of the microwells was produced using the Fit Spline modeling of GraphPad Prism Version 9.02 (GraphPad Software, California, United States).

## Data Availability

The raw data supporting the conclusion of this article will be made available by the authors, without undue reservation.

## References

[B1] Abbott J. Ye T. Krenek K. Qin L. Kim Y. Wu W. (2020). The design of a CMOS nanoelectrode array with 4096 current-clamp/voltage-clamp amplifiers for intracellular recording/stimulation of mammalian neurons. IEEE J. Solid-State Circuits 55 (9), 2567–2582. 10.1109/jssc.2020.3005816 33762776 PMC7983016

[B2] Ahrens M. B. Orger M. B. Robson D. N. Li J. M. Keller P. J. (2013). Whole-brain functional imaging at cellular resolution using light-sheet microscopy. Nat. Methods 10 (5), 413–420. 10.1038/nmeth.2434 23524393

[B3] Centeno M. Carmichael D. W. (2014). Network connectivity in epilepsy: resting state fMRI and EEG-fMRI contributions. Front. Neurol. 5 (93), 93. 10.3389/fneur.2014.00093 25071695 PMC4081640

[B4] Cervia L. D. Yuan F. (2018). Current progress in electrotransfection as a nonviral method for gene delivery. Mol. Pharm. 15 (9), 3617–3624. 10.1021/acs.molpharmaceut.8b00207 29889538 PMC6123289

[B5] Chang C. C. Mao M. Liu Y. Wu M. Vo-Dinh T. Yuan F. (2016). Improvement in electrotransfection of cells using carbon-based electrodes. Cell. Mol. Bioeng. 9 (4), 538–545. 10.1007/s12195-016-0452-9 28239428 PMC5321229

[B6] Chen N-C. Chen C-H. Chen M-K. Jang L-S. Wang M-H. (2014). Single-cell trapping and impedance measurement utilizing dielectrophoresis in a parallel-plate microfluidic device. Sensors Actuators B Chem. 190, 570–577. 10.1016/j.snb.2013.08.104

[B7] Choi J. S. Lee H. J. Rajaraman S. Kim D. H. (2021). Recent advances in three-dimensional microelectrode array technologies for *in vitro* and *in vivo* cardiac and neuronal interfaces. Biosens. Bioelectron. 171, 112687. 10.1016/j.bios.2020.112687 33059168 PMC7665982

[B8] Demirkaya A. K. Gundogdu G. Karakaya S. Taşci Ş. Y. Nalci K. A. Hacimüftüoğlu A. (2021). Does umbelliferone protect primary cortical neuron cells against glutamate excitotoxicity? Kafkas Univ. Veteriner Fak. Derg. 10.9775/kvfd.2021.25439

[B9] Dubey A. Ray S. (2019). Cortical electrocorticogram (ECoG) is a local signal. J. Neurosci. 39 (22), 4299–4311. 10.1523/jneurosci.2917-18.2019 30914446 PMC6538865

[B10] Farasat M. Chavoshi S. M. Bakhshi A. Valipour A. Badieirostami M. (2021). A dielectrophoresis-based microfluidic chip for trapping circulating tumor cells using a porous membrane. J. Micromechanics Microengineering 32 (1), 015008. 10.1088/1361-6439/ac3c89

[B11] Geraili A. Jafari P. Hassani M. S. Araghi B. H. Mohammadi M. H. Ghafari A. M. (2018). Controlling differentiation of stem cells for developing personalized organ-on-chip platforms. Adv. Healthc. Mater. 7 (2), 1700426. 10.1002/adhm.201700426 28910516

[B12] Golyala A. Kwan P. (2017). Drug development for refractory epilepsy: the past 25 years and beyond. Seizure*.* 44, 147–156. 10.1016/j.seizure.2016.11.022 28017578

[B13] Guan R. Chen Y. Zeng L. Rees T. W. Jin C. Huang J. (2018). Oncosis-inducing cyclometalated iridium(iii) complexes. Chem. Sci. 9 (23), 5183–5190. 10.1039/c8sc01142g 29997872 PMC6000986

[B14] Gupta P. Balasubramaniam N. Chang H. Y. Tseng F. G. Santra T. S. (2020). A single-neuron: current trends and future prospects. Cells 9 (6), 9061528. 10.3390/cells9061528 PMC7349798 32585883

[B15] Haque M. R. Wessel C. R. Leary D. D. Wang C. Bhushan A. Bishehsari F. (2022). Patient-derived pancreatic cancer-on-a-chip recapitulates the tumor microenvironment. Microsyst. Nanoeng. 8, 36. 10.1038/s41378-022-00370-6 35450328 PMC8971446

[B16] Heida T. (2003). Electric field-induced effects on neuronal cell biology accompanying dielectrophoretic trapping - introduction. Electr. Field-Induced Eff. Neuronal Cell Biol. Accompanying Dielectrophor. Trapp. 173, 1. 10.1007/978-3-642-55469-8_1 12901336

[B17] Holloway P. M. Willaime-Morawek S. Siow R. Barber M. Owens R. M. Sharma A. D. (2021). Advances in microfluidic *in vitro* systems for neurological disease modeling. J. Neurosci. Res. 99 (5), 1276–1307. 10.1002/jnr.24794 33583054

[B18] Huang S. He Y-Q. Jiao F. (2017). Advances of particles/cells magnetic manipulation in microfluidic chips. Chin. J. Anal. Chem. 45 (8), 1238–1246. 10.1016/s1872-2040(17)61033-8

[B19] Ji B. Liang Z. Yuan X. Xu H. Wang M. Yin E. (2022). Recent advances in wireless epicortical and intracortical neuronal recording systems. Sci. China Inf. Sci. 65 (4), 140401–140418. 10.1007/s11432-021-3373-1

[B20] Kajtez J. Buchmann S. Vasudevan S. Birtele M. Rocchetti S. Pless C. J. (2020). 3D-Printed soft lithography for complex compartmentalized microfluidic neural devices. Adv. Sci. 7 (16), 2001150. 10.1002/advs.202001150 PMC7435242 32832365

[B21] Kassab A. Le Lan J. Tremblay J. Vannasing P. Dehbozorgi M. Pouliot P. (2018). Multichannel wearable fNIRS-EEG system for long-term clinical monitoring. Hum. Brain Mapp. 39 (1), 7–23. 10.1002/hbm.23849 29058341 PMC6866376

[B22] Konishi S. Fujita T. Hattori K. Kono Y. Matsushita Y. (2015). An openable artificial intestinal tract system for the *in vitro* evaluation of medicines. Microsyst. Nanoeng. 1, 15015. 10.1038/micronano.2015.15

[B23] Kumar S. S. Baker M. S. Okandan M. Muthuswamy J. (2020). Engineering microscale systems for fully autonomous intracellular neural interfaces. Microsyst. Nanoeng. 6 (1), 1. 10.1038/s41378-019-0121-y 34567616 PMC8433365

[B24] Li Y. M. Jin J. S. Bai F. (2022). Cancer biology deciphered by single-cell transcriptomic sequencing. Protein & Cell 13 (3), 167–179. 10.1007/s13238-021-00868-1 34405376 PMC8901819

[B25] Liang Y. Offenhausser A. Ingebrandt S. Mayer D. (2021). PEDOT:PSS-Based bioelectronic devices for recording and modulation of electrophysiological and biochemical cell signals. Adv. Healthc. Mater. 10 (11), e2100061. 10.1002/adhm.202100061 33970552 PMC11468774

[B26] Luo T. Fan L. Zhu R. Sun D. (2019). Microfluidic single-cell manipulation and analysis: methods and applications. Micromachines (Basel) 10 (2), 10020104. 10.3390/mi10020104 PMC6412357 30717128

[B27] Morales-Carvajal P. M. Kundu A. Didier C. M. Hart C. Sommerhage F. Rajaraman S. (2020). Makerspace microfabrication of a stainless steel 3D microneedle electrode array (3D MEA) on a glass substrate for simultaneous optical and electrical probing of electrogenic cells. RSC Adv. 10 (68), 41577–41587. 10.1039/d0ra06070d 35516576 PMC9057996

[B28] Osaki T. Shin Y. Sivathanu V. Campisi M. Kamm R. D. (2018). *In vitro* microfluidic models for neurodegenerative disorders. Adv. Healthc. Mater. 7 (2), 1700489. 10.1002/adhm.201700489 28881425

[B29] Pang L. Ding J. Liu X-X. Yuan H. Ge Y. Fan J. (2020). Microstructure-based techniques for single-cell manipulation and analysis. TrAC Trends Anal. Chem. 129, 115940. 10.1016/j.trac.2020.115940

[B30] Pelkonen A. Mzezewa R. Sukki L. Ryynänen T. Kreutzer J. Hyvärinen T. (2020). A modular brain-on-a-chip for modelling epileptic seizures with functionally connected human neuronal networks. Biosens. Bioelectron. 168, 112553. 10.1016/j.bios.2020.112553 32877779

[B31] Saiding Q. Ma J. Ke C. Cui W. (2022). From "organs on a chip" to "patient on a chip". Innov. (Camb) 3 (5), 100282. 10.1016/j.xinn.2022.100282 PMC9307665 35880236

[B32] Seok J. Warren H. S. Cuenca A. G. Mindrinos M. N. Baker H. V. Xu W. (2013). Genomic responses in mouse models poorly mimic human inflammatory diseases. Proc. Natl. Acad. Sci. U. S. of America. 110 (9), 3507–3512. 10.1073/pnas.1222878110 PMC3587220 23401516

[B33] Tang F. Hartz A. M. S. Bauer B. (2017). Drug-resistant epilepsy: multiple hypotheses, few answers. Front. Neurol. 8, 301. 10.3389/fneur.2017.00301 28729850 PMC5498483

[B34] Tsai D. Sawyer D. Bradd A. Yuste R. Shepard K. L. (2017). A very large-scale microelectrode array for cellular-resolution electrophysiology. Nat. Commun. 8 (1), 1802. 10.1038/s41467-017-02009-x 29176752 PMC5702607

[B35] Wijdenes P. Ali H. Armstrong R. Zaidi W. Dalton C. Syed N. I. (2016). A novel bio-mimicking, planar nano-edge microelectrode enables enhanced long-term neural recording. Sci. Rep. 6, 34553. 10.1038/srep34553 27731326 PMC5059639

[B36] Wu C. Chen R. Liu Y. Yu Z. Jiang Y. Cheng X. (2017). A planar dielectrophoresis-based chip for high-throughput cell pairing. Lab a Chip. 17 (23), 4008–4014. 10.1039/c7lc01082f 29115319

[B37] Xu D. X. Fang J. R. Zhang M. Y. Wang H. Zhang T. Hang T. (2021). Synchronized intracellular and extracellular recording of action potentials by three-dimensional nanoroded electroporation. Biosens. Bioelectron. 192, 113501. 10.1016/j.bios.2021.113501 34273736

[B38] Yang Y. Pang W. Zhang H. Cui W. Jin K. Sun C. (2022). Manipulation of single cells via a stereo acoustic streaming tunnel (SteAST). Microsyst. Nanoeng. 8, 88. 10.1038/s41378-022-00424-9 35935274 PMC9352906

[B39] Yao J. Zhu G. Zhao T. Takei M. (2019). Microfluidic device embedding electrodes for dielectrophoretic manipulation of cells-A review. Electrophoresis 40, 1166–1177. 10.1002/elps.201800440 30378130

[B40] Yoshida S. Teshima T. Kuribayashi-Shigetomi K. Takeuchi S. (2016). Mobile microplates for morphological control and assembly of individual neural cells. Adv. Healthc. Mater. 5 (4), 415–420. 10.1002/adhm.201500782 26712104

[B41] Zhang H. Rong G. Bian S. Sawan M. (2022). Lab-on-Chip microsystems for *ex vivo* network of neurons studies: A review. Front. Bioeng. Biotechnol. 10, 841389. 10.3389/fbioe.2022.841389 35252149 PMC8888888

