# Supplementary material for: Single neurons on microelectrode array chip: manipulation and analyses
Source: Front Bioeng Biotechnol. 2023 Sep 27;11:1258626. doi: 10.3389/fbioe.2023.1258626 (PMC10565505; doi:10.3389/fbioe.2023.1258626)
Supplement: Supplementary file 3 [file DataSheet1.docx]

**Supplementary Materials for**

**Single Neurons on Microelectrode Array Chip: Manipulation and Analyses**

Hongyong Zhang^a,b^, Pengbo Wang^b^, Nan Huang^c^, Lingrui Zhao^c^, Yi Su^b^, Lingfei Li^d^, Sumin Bian^b,^***, and Mohamad Sawan^b,^***

*^a^ Zhejiang University, Hangzhou, 310058, China*

*^b^ Research Center for Industries of the Future, Westlake University, Hangzhou, Zhejiang 310030, China*

*^c^ School of Life Science,* *Westlake University, Hangzhou, 310024, China*

*^d^ Department of Neurology, Affiliated Hangzhou First People’s Hospital, Zhejiang University School of Medicine, 310003, China*

** Corresponding authors: biansumin@westlake.edu.cn;* [*sawan@westlake.edu.cn*](mailto:sawan@westlake.edu.cn)

Supplemental information includes nine figures.


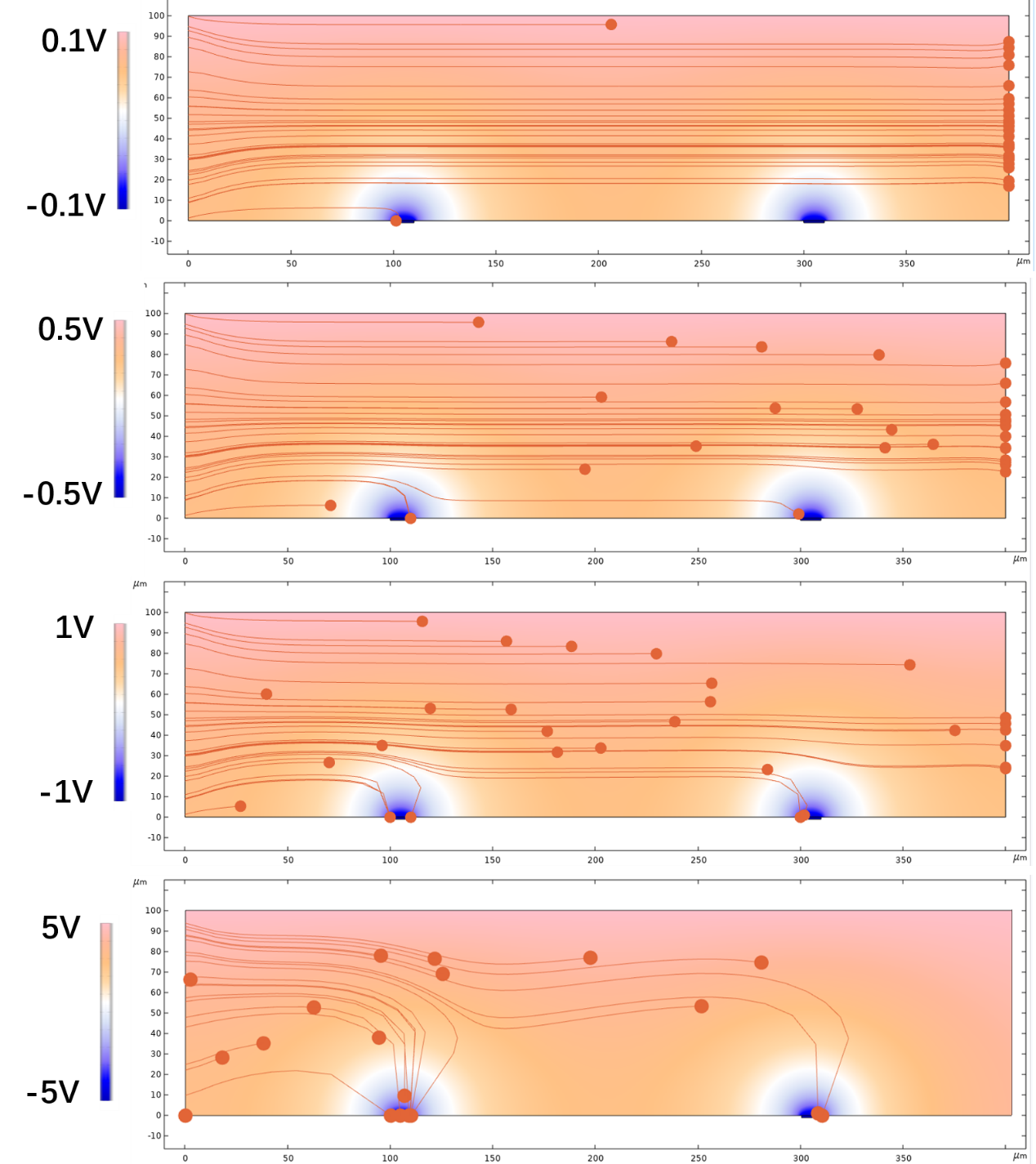


**Fig. S1**. Simulation results by COMSOL of cell manipulation under different voltage levels, from 0.2V to 10V. Cell trajectories indicated that increasing the voltage enhanced dielectrophoresis force and captured more particles.


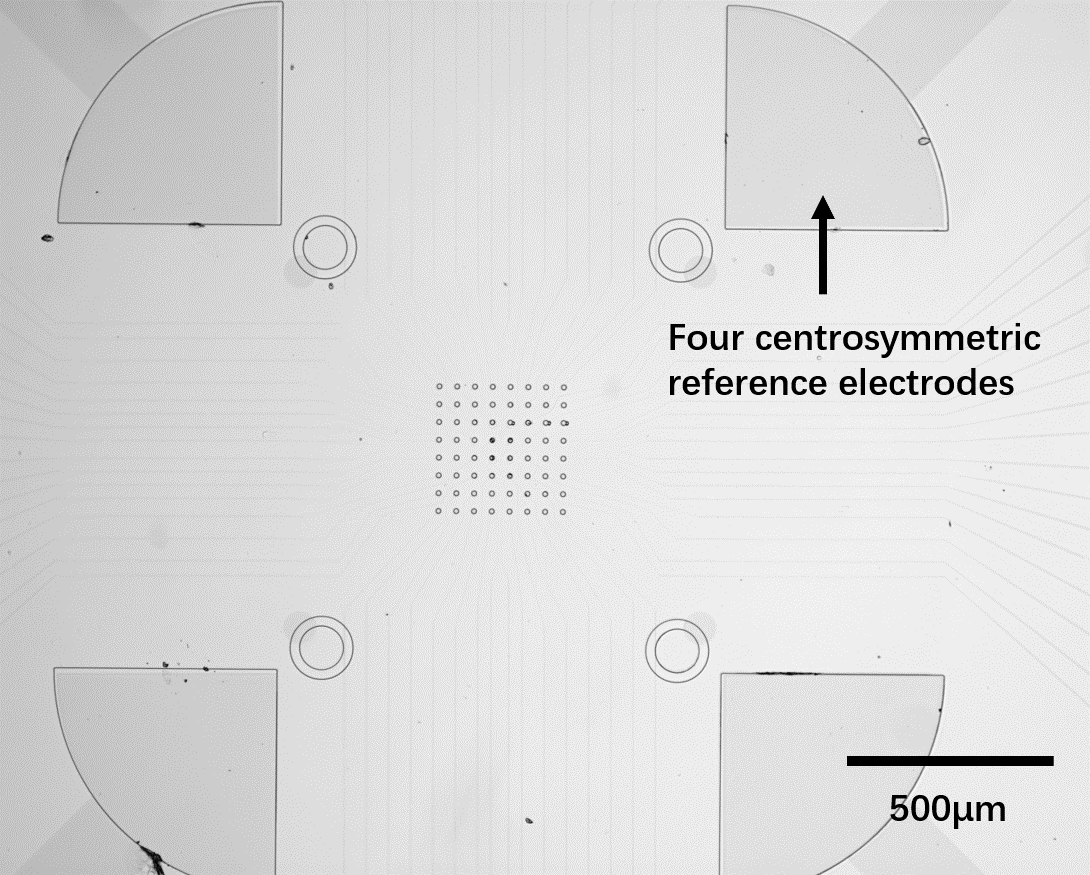


**Fig. S2.** Position of four reference electrodes relative to the microelectrodes. (Scale bar: 1000 μm)


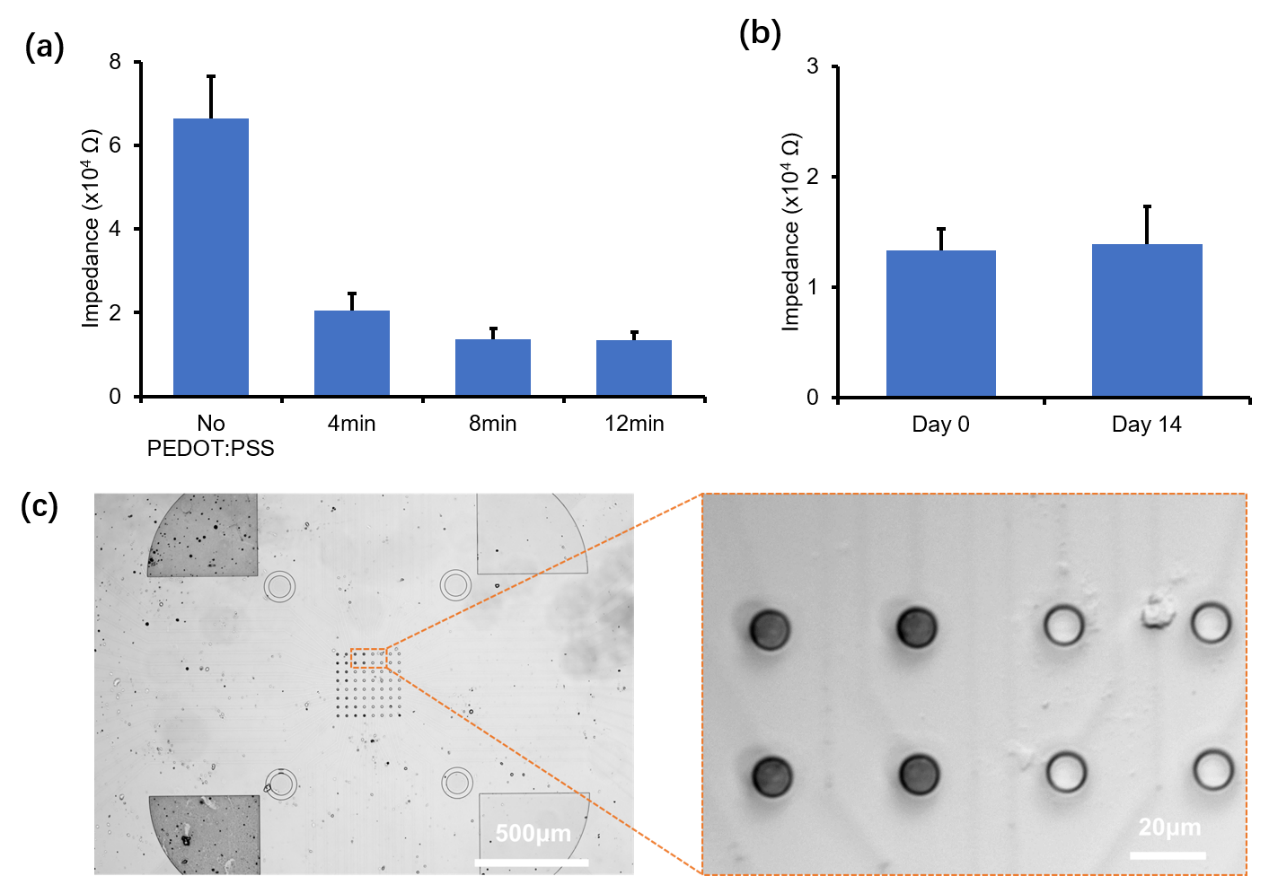


**Fig. S3.** Electrodeposition characteristics of PEDOT: PSS: (a) Impedance of the microelectrodes with and without PEDOT:PSS coating; (b) The electrodes were immersed in culture medium for two weeks and the impedance maintained in a low level; (c) Parts of the electrodes on one chip was coated with PEDOT:PSS and the difference is obvious.


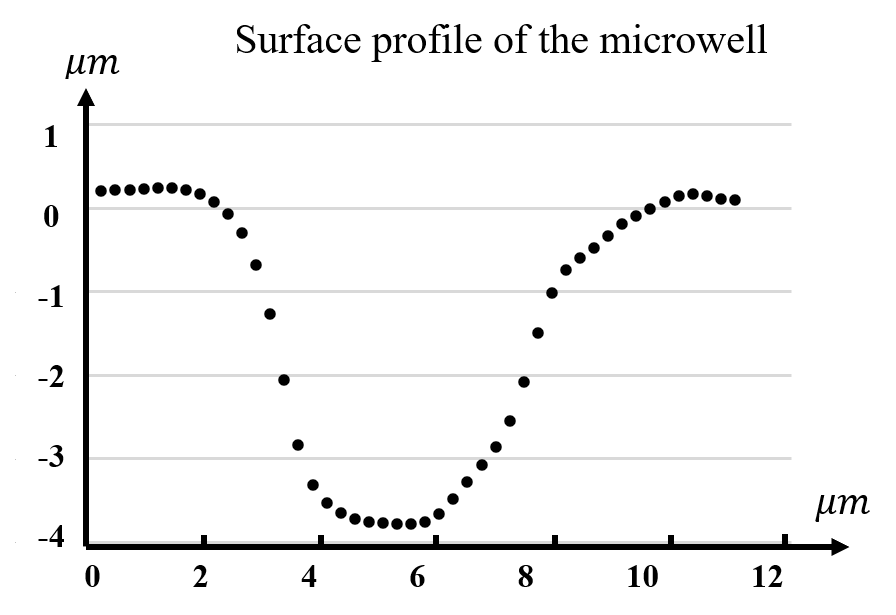


**Fig. S4.** The surface profile of the microwells measured by the surface profile measuring system. Data exhibited the depth of the hole at approximately 4 µm, which was smaller than the average radii of 293T and neural cells.


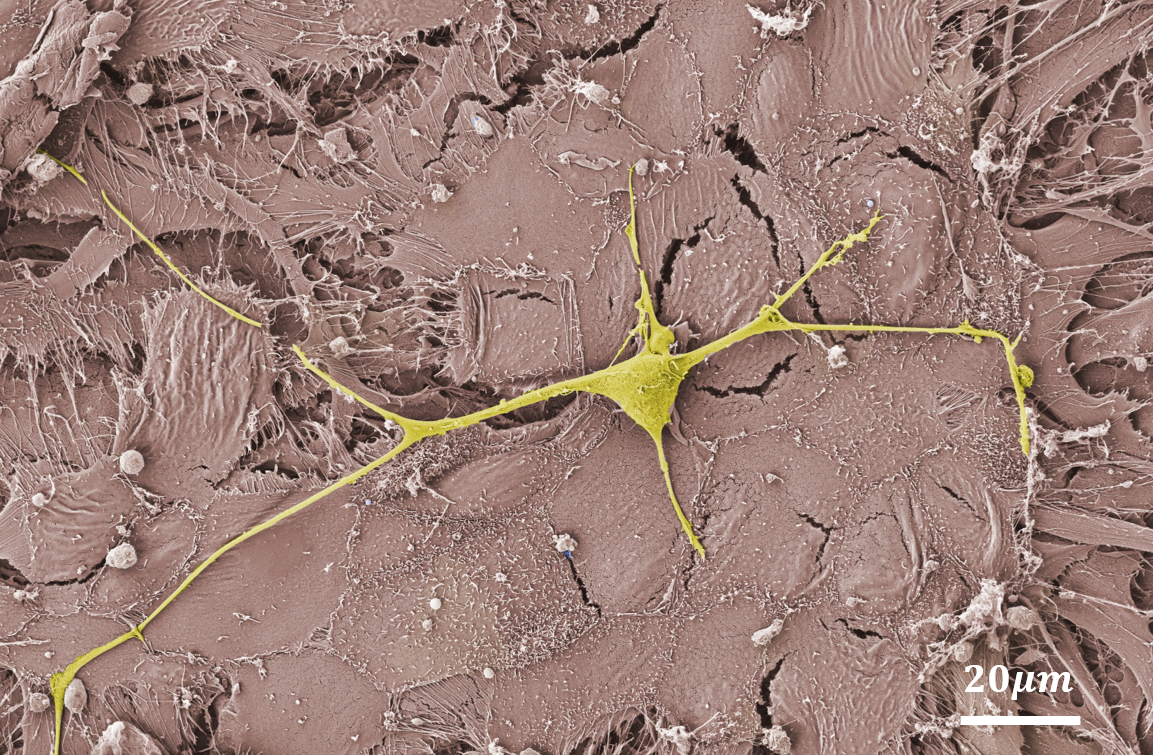


**Fig. S5.** SEM image of a single neuron. The morphology of the neuron is quite clear (yellow). The Matrigel base cracked due to dehydration and critical point dry (brown).


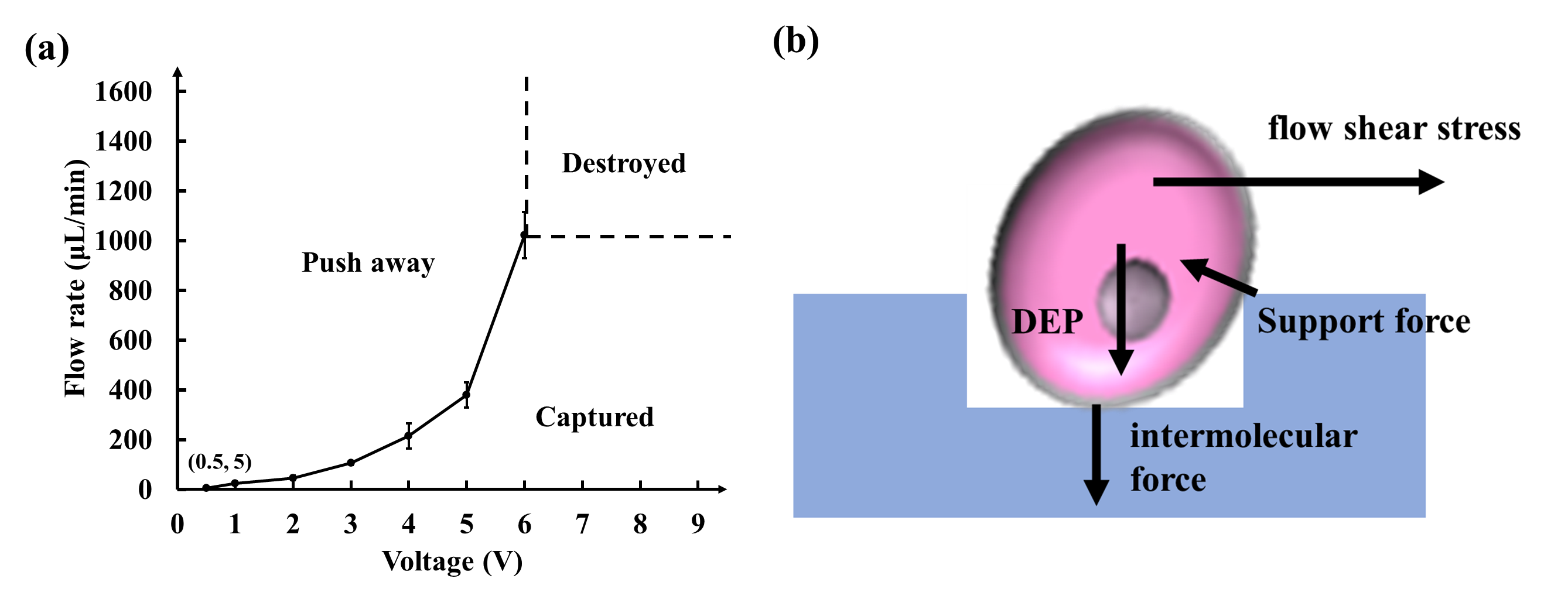


**Fig. S6.** (a) The states of cells under different voltage and flow rate; (b) Force analysis diagram of trapped single cell.


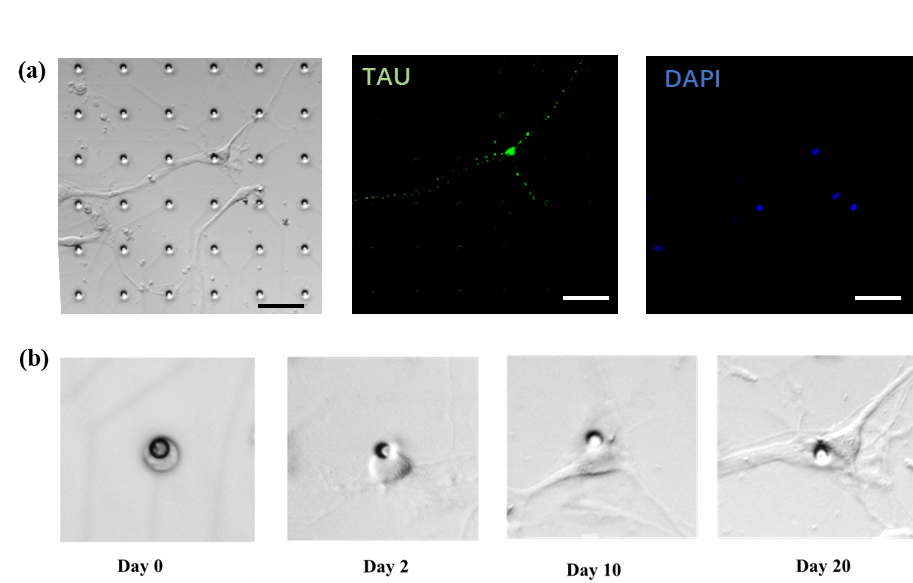


**Fig. S7.** (a) Fluorescent images of single neurons on chip (TAU and DAPI) to exhibit the morphology of neurons. (Scale bar = 50μm); (b) Growing process of single cell on electrodes in representative stage.


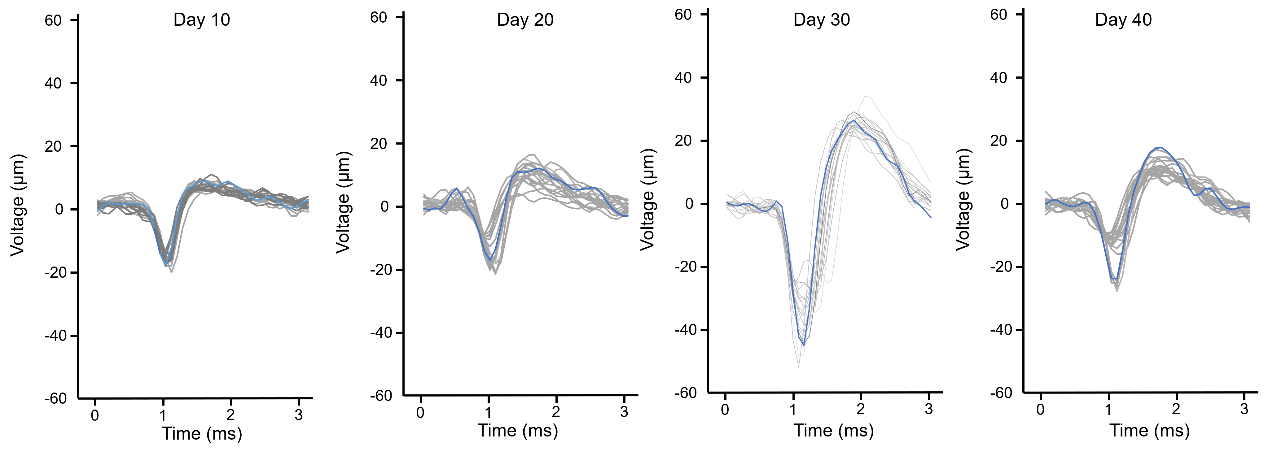


**Fig. S8.** Typical overlaid spike waveform from NoNs in different culture days. The spike amplitudes of NoNs increased with culturing time and reached a maximum on day 30.


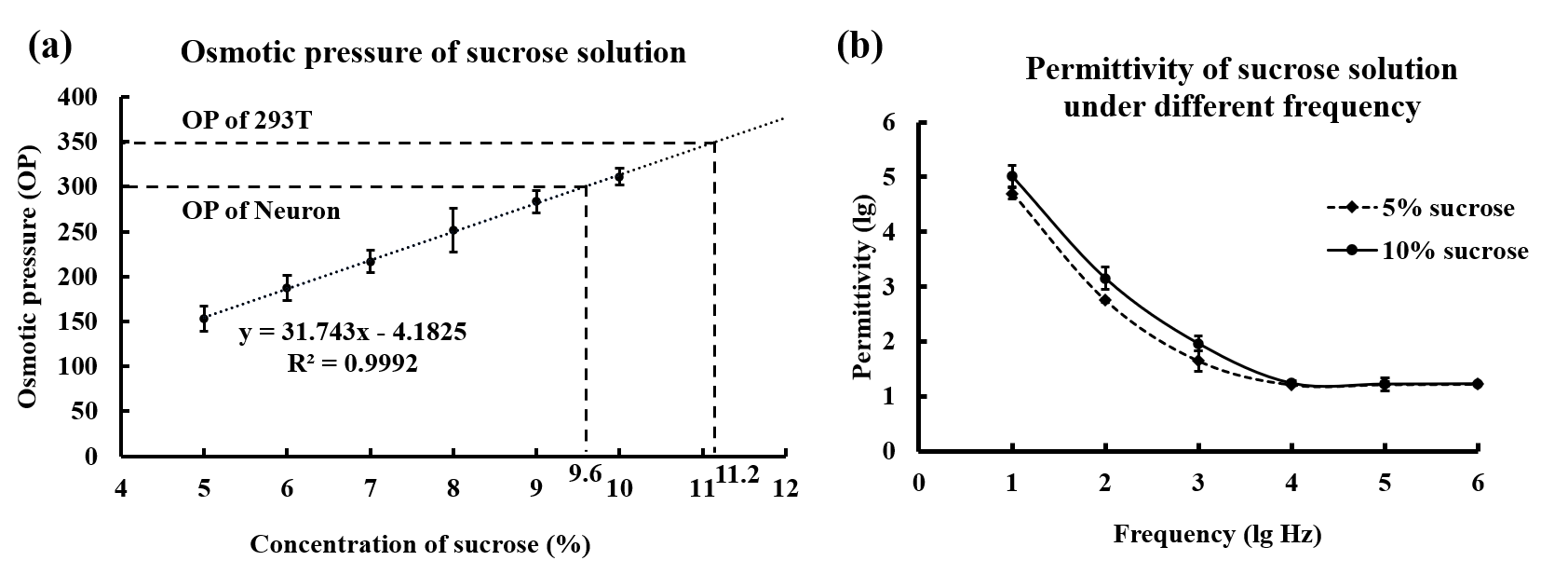


**Fig. S9.** The properties of sucrose solution: (a) The osmotic pressure (OP) of sucrose solution with different concentrations. Compared with the OP of cell culture medium, we could determine the suitable concentration for different cells; (b) The permittivity of sucrose solution under different measure frequency.


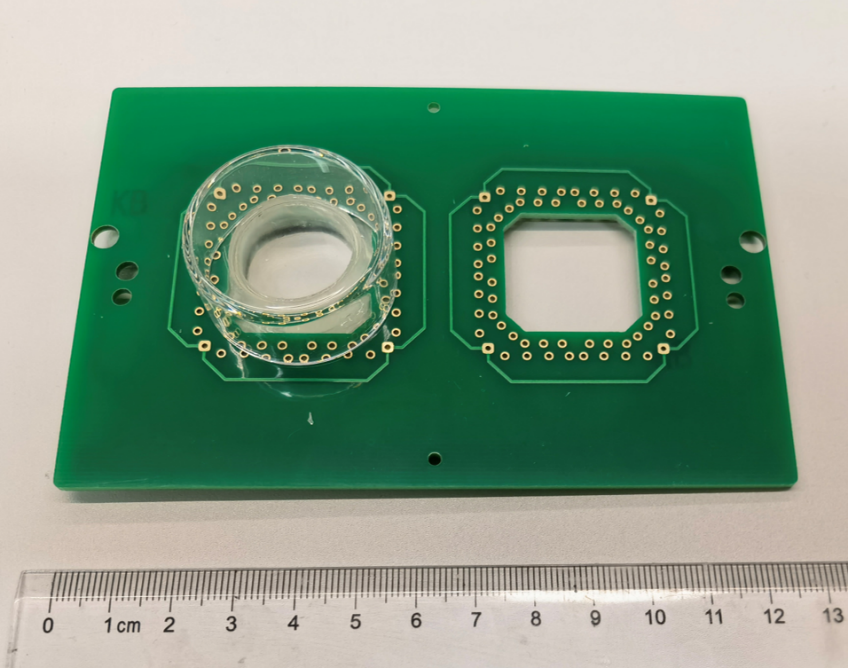


**Fig. S10.** The matched PCB was designed to connect our MEA chip with the commercial data acquisition system.
